# Galactorrhoea as a side effect due to Bupropion- a case report

**DOI:** 10.1192/j.eurpsy.2021.2225

**Published:** 2021-08-13

**Authors:** D. Bhandutia, S. Nayok

**Affiliations:** 1 Psychiatry, Sri Siddhartha Medical College, Tumakuru, India; 2 Psychiatry, Sri Siddhartha Medical College, Tumkur, India

**Keywords:** Bupropion, Galactorrhoea

## Abstract

**Introduction:**

Bupropion is a NDRI antidepressant with action on both serotonin and nicotinic receptors. Endocrine and sexual adverse effects are very rare and hence very unlikely to cause hyperprolactinemia. We report a case of a patient who developed galactorrhoea following Bupropion augmentation of Escitalopram. A 24 yr old unmarried nulliparous female was brought with complaints of low mood, loss of interest, decreased concentration in studies from 20 days. She was also reported to be smoking cigarettes since 2 years with occasional alcohol use. There was no menstrual abnormalities nor any use of regular medication. On MSE there was depressed affect with negative cognition and occasional death wishes with normal perception. HAM-D Score was 17-19. She was started on Escitalopram 10 mg/day and Clonazepam 0.5 mg/day. Depressive symptoms improved and 2 weeks later Bupropion 150 mg/day was added as anti-craving and for augmentation owing to residual depressive symptoms.

**Objectives:**

Bupropion induced Galactorrhoea

**Methods:**

Cross-sectional

**Results:**

Within 2 weeks of starting Bupropion, she reported with complaint of galactorrhoea. Prolactin level came out to be 28.67 ug/L. Brain imaging was also reported to be normal. Escitalopram was stopped and substituted with Mirtazapine 7.5 mg/day, continued for a week. There was no improvement, hence Mirtazapine and Bupropion were discontinued and started on Sertraline 25 mg/day. Galactorrhoea started reducing within next 4-5 days and completely subsided within a week of stopping Bupropion.

**Conclusions:**

Bupropion has action on serotonin receptors which might be hypothesized as cause. Also, it is a potent CYP2D6 inhibitor, causing increase levels of Escitalopram.

**Disclosure:**

No significant relationships.

